# Supplementary material for: Contributions of the Left and the Right Hemispheres on Language-Induced Grip Force Modulation of the Left Hand in Unimanual Tasks
Source: Medicina (Kaunas). 2019 Oct 6;55(10):674. doi: 10.3390/medicina55100674 (PMC6843471; doi:10.3390/medicina55100674)
Supplement: Supplementary file 1 [file medicina-55-00674-s001.zip › Contributions Left Right - Table S1 - Playlists.pdf]

**PLAYLISTS**

Playlists’ word sequence, by block. Total number of repetitions by block in brackets.

| | AMARRAR – TO TIE | |
|---|---|---|
| | BLOCK 1 *[11]* | BLOCK 2 *[7]* |
| **1** | Tábua | Pato |
| **2** | Sapo | Mala |
| **3** | Mamãe | *Amarrar* |
| **4** | Água | Rato |
| **5** | Carro | Faca |
| **6** | *Amarrar* | Escola |
| **7** | Tatu | Carro |
| **8** | *Amarrar* | Bola |
| **9** | Aranha | *Amarrar* |
| **10** | Repolho | Abraço |
| **11** | *Amarrar* | Estrela |
| **12** | Sapato | Apito |
| **13** | Estrela | Tatu |
| **14** | Lanche | *Amarrar* |
| **15** | *Amarrar* | Avião |
| **16** | Urso | Antena |
| **17** | Rato | *Amarrar* |
| **18** | *Amarrar* | Aranha |
| **19** | Escola | Jiló |
| **20** | Jogo | Mamãe |
| **21** | Pato | Água |
| **22** | Prego | *Amarrar* |
| **23** | *Amarrar* | Lanche |
| **24** | Caderno | Jogo |
| **25** | Bolsa | Tábua |
| **26** | *Amarrar* | Bolsa |
| **27** | Jiló | *Amarrar* |
| **28** | Abraço | Repolho |
| **29** | Mala | Sapo |
| **30** | Apito | Papai |
| **31** | *Amarrar* | Prego |
| **32** | Antena | Caderno |
| **33** | Dominó | Dominó |
| **34** | *Amarrar* | Sapato |
| **35** | Bola | *Amarrar* |
| **36** | *Amarrar* | Urso |
| **37** | Avião | Leite |
| **38** | Papai | |
| **39** | *Amarrar* | |
| **40** | Faca | |
| **41** | Leite | |

| | DIRIGIR – TO DRIVE | |
|---|---|---|
| | BLOCK 1 *[8]* | BLOCK 2 *[10]* |
| **1** | Repolho | Sapo |
| **2** | Estrela | Dominó |
| **3** | Mala | Mala |
| **4** | *Dirigir* | *Dirigir* |
| **5** | Jogo | Bolsa |
| **6** | Dominó | Jogo |
| **7** | Faca | *Dirigir* |
| **8** | Bola | Repolho |
| **9** | *Dirigir* | *Dirigir* |
| **10** | Pato | Carro |
| **11** | Tatu | Pato |
| **12** | Abraço | Papai |
| **13** | Bolsa | *Dirigir* |
| **14** | *Dirigir* | Tábua |
| **15** | Carro | Rato |
| **16** | Jiló | Leite |
| **17** | Prego | *Dirigir* |
| **18** | *Dirigir* | Sapato |
| **19** | Avião | Estrela |
| **20** | Papai | Caderno |
| **21** | *Dirigir* | Prego |
| **22** | Rato | *Dirigir* |
| **23** | Leite | Abraço |
| **24** | Sapato | Bola |
| **25** | Sapo | Antena |
| **26** | Urso | Avião |
| **27** | Tábua | *Dirigir* |
| **28** | *Dirigir* | Aranha |
| **29** | Caderno | Apito |
| **30** | Mamãe | *Dirigir* |
| **31** | Aranha | Lanche |
| **32** | Lanche | Mamãe |
| **33** | *Dirigir* | Escola |
| **34** | Antena | Jiló |
| **35** | Apito | *Dirigir* |
| **36** | *Dirigir* | Urso |
| **37** | Escola | Água |
| **38** | Água | *Dirigir* |
| **39** | | Tatu |
| **40** | | Faca |
| **41** | | |

## PLAYLISTS

Playlists’ word sequence, by block. Total number of repetitions by block in brackets.

| | ESCREVER – TO WRITE | |
|---|---|---|
| | **BLOCK 1** *[9]* | **BLOCK 2** *[9]* |
| **1** | Estrela | Bolsa |
| **2** | *Escrever* | *Escrever* |
| **3** | Papai | Rato |
| **4** | Apito | Bola |
| **5** | Lanche | *Escrever* |
| **6** | Avião | Aranha |
| **7** | Antena | Carro |
| **8** | Jiló | Apito |
| **9** | Repolho | Mamãe |
| **10** | Dominó | Faca |
| **11** | *Escrever* | Dominó |
| **12** | Leite | *Escrever* |
| **13** | Bolsa | Mala |
| **14** | Mamãe | Tábua |
| **15** | Água | Sapato |
| **16** | *Escrever* | *Escrever* |
| **17** | Tatu | Estrela |
| **18** | *Escrever* | Escola |
| **19** | Sapato | Pato |
| **20** | *Escrever* | *Escrever* |
| **21** | Escola | Sapo |
| **22** | *Escrever* | Repolho |
| **23** | Carro | Tatu |
| **24** | Mala | *Escrever* |
| **25** | Bola | Jiló |
| **26** | Aranha | Papai |
| **27** | *Escrever* | Caderno |
| **28** | Caderno | *Escrever* |
| **29** | Faca | Leite |
| **30** | Tábua | Antena |
| **31** | *Escrever* | Prego |
| **32** | Sapo | *Escrever* |
| **33** | *Escrever* | Avião |
| **34** | Rato | *Escrever* |
| **35** | Prego | Água |
| **36** | Pato | Lanche |
| **37** | | |
| **38** | | |
| **39** | | |
| **40** | | |
| **41** | | |

| | DESENHAR – TO DRAW | |
|---|---|---|
| | **BLOCK 1** *[7]* | **BLOCK 2** *[11]* |
| **1** | Aranha | Prego |
| **2** | *Desenhar* | Mala |
| **3** | Bola | *Desenhar* |
| **4** | *Desenhar* | Carro |
| **5** | Leite | Tábua |
| **6** | Tábua | *Desenhar* |
| **7** | Bolsa | Apito |
| **8** | Pato | Sapo |
| **9** | Faca | *Desenhar* |
| **10** | *Desenhar* | Sapato |
| **11** | Avião | Caderno |
| **12** | Mamãe | *Desenhar* |
| **13** | Antena | Rato |
| **14** | Lanche | Avião |
| **15** | Sapo | Tatu |
| **16** | Estrela | Aranha |
| **17** | Dominó | Antena |
| **18** | *Desenhar* | *Desenhar* |
| **19** | Repolho | Água |
| **20** | Apito | Estrela |
| **21** | *Desenhar* | Escola |
| **22** | Escola | *Desenhar* |
| **23** | Tatu | Repolho |
| **24** | Água | *Desenhar* |
| **25** | Sapato | Dominó |
| **26** | Papai | Mamãe |
| **27** | *Desenhar* | *Desenhar* |
| **28** | Carro | Pato |
| **29** | Caderno | Bolsa |
| **30** | Jiló | Jiló |
| **31** | Mala | Papai |
| **32** | Rato | *Desenhar* |
| **33** | *Desenhar* | Faca |
| **34** | Prego | *Desenhar* |
| **35** | | Leite |
| **36** | | Bola |
| **37** | | *Desenhar* |
| **38** | | Lanche |
| **39** | | |
| **40** | | |
| **41** | | |

**PLAYLISTS**

Playlists’ word sequence, by block. Total number of repetitions by block in brackets.

| | **SEGURAR – TO HOLD** | |
|---|---|---|
| | **BLOCK 1** *[9]* | **BLOCK 2** *[9]* |
| **1** | Mala | Dominó |
| **2** | Avião | Pato |
| **3** | *Segurar* | Sapo |
| **4** | Sapo | *Segurar* |
| **5** | Jiló | Repolho |
| **6** | Sapato | Escola |
| **7** | *Segurar* | *Segurar* |
| **8** | Jogo | Bola |
| **9** | Faca | Lanche |
| **10** | Dominó | *Segurar* |
| **11** | Papai | Jogo |
| **12** | Apito | *Segurar* |
| **13** | *Segurar* | Leite |
| **14** | Escola | Bolsa |
| **15** | Urso | Faca |
| **16** | Estrela | Avião |
| **17** | *Segurar* | Tábua |
| **18** | Bolsa | *Segurar* |
| **19** | Tábua | Mamãe |
| **20** | *Segurar* | Urso |
| **21** | Carro | Carro |
| **22** | Bola | Rato |
| **23** | Aranha | Aranha |
| **24** | Abraço | *Segurar* |
| **25** | *Segurar* | Mala |
| **26** | Leite | Apito |
| **27** | Mamãe | *Segurar* |
| **28** | Lanche | Papai |
| **29** | *Segurar* | *Segurar* |
| **30** | Repolho | Tatu |
| **31** | Rato | Prego |
| **32** | Tatu | Sapato |
| **33** | *Segurar* | Jiló |
| **34** | Prego | Abraço |
| **35** | Antena | *Segurar* |
| **36** | *Segurar* | Caderno |
| **37** | Pato | Estrela |
| **38** | Água | Antena |
| **39** | Caderno | Água |
| **40** | | |
| **41** | | |

| | **PUXAR – TO PULL** | |
|---|---|---|
| | **BLOCK 1** *[10]* | **BLOCK 2** *[8]* |
| **1** | Repolho | Caderno |
| **2** | Avião | Jogo |
| **3** | Apito | Urso |
| **4** | Leite | Prego |
| **5** | *Puxar* | *Puxar* |
| **6** | Tábua | Repolho |
| **7** | Carro | Mala |
| **8** | *Puxar* | Faca |
| **9** | Sapo | *Puxar* |
| **10** | *Puxar* | Leite |
| **11** | Papai | Sapo |
| **12** | Abraço | *Puxar* |
| **13** | Sapato | Carro |
| **14** | Tatu | Apito |
| **15** | *Puxar* | Escola |
| **16** | Antena | Água |
| **17** | Estrela | *Puxar* |
| **18** | Bolsa | Tatu |
| **19** | *Puxar* | Dominó |
| **20** | Rato | Avião |
| **21** | *Puxar* | Tábua |
| **22** | Jogo | *Puxar* |
| **23** | Aranha | Bolsa |
| **24** | Caderno | Sapato |
| **25** | Lanche | *Puxar* |
| **26** | *Puxar* | Lanche |
| **27** | Água | Abraço |
| **28** | Urso | Rato |
| **29** | Jiló | *Puxar* |
| **30** | *Puxar* | Bola |
| **31** | Dominó | Antena |
| **32** | Bola | Estrela |
| **33** | Escola | Jiló |
| **34** | Faca | Papai |
| **35** | Mala | *Puxar* |
| **36** | *Puxar* | Aranha |
| **37** | Mamãe | Pato |
| **38** | Pato | Mamãe |
| **39** | *Puxar* | |
| **40** | Prego | |
| **41** | | |
