# Supplementary material for: Contributions of the Left and the Right Hemispheres on Language-Induced Grip Force Modulation of the Left Hand in Unimanual Tasks
Source: Medicina (Kaunas). 2019 Oct 6;55(10):674. doi: 10.3390/medicina55100674 (PMC6843471; doi:10.3390/medicina55100674)
Supplement: Supplementary file 1 [file medicina-55-00674-s001.zip › Contributions Left Right - Table S2 - Word list.pdf]

Word list

Portuguese nouns and verbs utilised in this research and its equivalents in English

n	Portuguese verbs	English verbs	n	Portuguese verbs	English verbs
1	Desenhar	To draw	4	Puxar	To pull
2	Dirigir	To drive	5	Segurar	To hold
3	Escrever	To write	6	Amarrar	To tie

n	Portuguese nouns	English nouns	n	Portuguese nouns	English nouns
1	Antena	Antenna	19	Estrela	Star
2	Aranha	Spider	20	Giló	Giloh
3	Avião	Avion	21	Sapo	Frog
4	Mala	Suitcase	22	Brinquedo	Toy
5	Bola	Ball	23	Leite	Milk
6	Bolsa	Bag	24	Mamãe	Mother
7	Caderno	Notebook	25	Relógio	Clock
8	Pato	Duck	26	Urso	Bear
9	Sapato	Shoe	27	Papai	Father
10	Repolho	Cabbage	28	Pé	Foot
11	Prego	Nail	29	Tábua	Board
12	Lanche	Snack	30	Reunião	Meeting
13	Domingo	Sunday	31	Mochila	Schoolbag
14	Dominó	Domino	32	Apito	Whistle
15	Água	Water	33	Rato	Mouse
16	Escola	School	34	Tatu	Armadillo
17	Abraço	Hug	35	Carro	Car
18	Escada	Ladder			
